# Molecular characterization of *Blastocystis* subtypes in HIV-positive patients and evaluation of risk factors for colonization

**DOI:** 10.1186/s12879-019-4537-7

**Published:** 2019-10-22

**Authors:** Lucia Fontanelli Sulekova, Simona Gabrielli, Federica Furzi, Giovanni Luigi Milardi, Elisa Biliotti, Maurizio De Angelis, Giancarlo Iaiani, Caterina Fimiani, Myriam Maiorano, Simonetta Mattiucci, Gloria Taliani

**Affiliations:** 1 Department of Translation and Precision Medicine, Sapienza University of Rome, 00185 Rome, Italy; 2 Clinical Diagnostic Parasitology laboratory, Umberto I Academic Hospital, 00185 Rome, Italy; 3 Department of Public Health and Infectious Diseases, Sapienza University of Rome, Piazzale Aldo Moro 5, 00185 Rome, Italy

**Keywords:** *Blastocystis*, Subtype, PCR, HIV, Italy

## Abstract

**Background:**

*Blastocystis* is one of the most common intestinal protozoa in human faecal samples with uncertain impact on public health*.* Studies on the prevalence of *Blastocystis* in HIV-positive patients are limited and dated.

**Methods:**

A cross-sectional study was carried out involving 156 HIV-positive patients to evaluate the prevalence of *Blastocystis*-subtypes by molecular amplification and sequencing the small subunit rRNA gene (SSU rDNA), to identify the risk factors for its transmission, to examine the relationship between the presence of the protist and gastrointestinal disorders. Furthermore, the evaluation of the faecal calprotectin by immunoassay from a sample of subjects was performed to evaluate the gut inflammation in *Blastocystis-*carriers.

**Results:**

*Blastocystis-*subtypes ST1, ST2, ST3, ST4 were identified in 39 HIV-positive patients (25%). No correlation was found between the presence of the protist and virological or epidemiological risk factors. *Blastocystis* was more frequently detected in homosexual subjects (*p* = 0.037) infected by other enteric protozoa (*p* = 0.0001) and with flatulence (*p* = 0.024). No significant differences in calprotectin level was found between *Blastocystis-*carriers and free ones.

**Conclusions:**

*Blastocystis* is quite common in HIV-positive patients on ART showing in examined patients 25% prevalence. Homosexual behaviour may represent a risk factor for its transmission, while CD4 count and viremia didn’t correlate with the presence of the protist. The pathogenetic role of *Blastocystis* remains unclear and no gut inflammation status was detected in *Blastocystis-*carriers. The only symptom associated with *Blastocystis* was the flatulence, evidencing a link between the presence of the protist and the composition and stability of gut microbiota.

## Background

*Blastocystis* spp. is a common intestinal protist distributed worldwide infecting humans and animals, with a prevalence from 0.5–30% and 30–76% in industrialized and developing countries, respectively [1]. This difference can be explained by poor hygiene practices and consumption of contaminated food or water [2–5] since the faecal-oral route is considered to be the main mode of transmission of this protist [6]. A remarkable genetic diversity has been revealed among *Blastocystis* spp. isolates from humans and animals based on the comparison of the small-subunit rRNA gene (SSU rDNA) sequences [7]*.* Currently at least 26 subtypes of *Blastocystis* have been described among mammalian and avian isolates [8, 9], nine of them (ST-1 to ST-8 and ST-12) detected in human population ﻿and are potentially zoonotic [10]. Because several STs are shared between humans and animals it has been proposed that a proportion of human infections may result from zoonotic transmission. Indeed, a higher risk of *Blastocystis* infection was found in people with close animal contact, including zookeepers [11]. Despite several studies reported *Blastocystis* implicated in different intestinal diseases, potential pathogenetic factors have been described and its presence is frequently associated with symptoms in humans [12], its pathogenetic role is so far under debated and ﻿several variables, as well as the *Blastocystis* subtype and load, host’s immune status and dysbiosis, could affect the occurrence of the disease [13, 14].

Although the protist was never considered as an opportunistic protozoon, it has also been frequently found in immunocompromised individuals presenting diarrhoea, with prevalence value ranging from 15 to 72.4% [15]. In addition, the sexual practices among men who have sex with men (MSM) have been reported to increase the transmission of *Blastocystis* (as for as other enteric organisms) [16]*.* Finally, recent data suggest that the protozoon is associated with certain gut microbiota profiles and health indices. Accordingly, a positive correlation between high bacterial richness and the presence of *Blastocystis* has been reported [17].

Notwithstanding it is commonly accepted that *Blastocystis* is a non-invasive organism, its vacuolar form is able to colonize the lamina propria, the submucosa and even the muscle layers leading to inflammation and active colitis in experimentally infected mice [18]. Currently, it is possible to perform a non-invasive evaluation of gut inflammation using biomarkers as the fecal calprotectin (FC), which is considered a surrogate marker of intestinal inflammation with good diagnostic performance for separating organic and functional intestinal disorders [19]*.* The increase of FC is due to faecal excretion of neutrophils and macrophages migrants from the bloodstream into the intestinal lumen, which occurs during the intestinal inflammation. The value of FC as a laboratory marker has been shown in inflammatory bowel disease [20] but its significance in other gastrointestinal diseases remains unclear. Studies evaluating the correlation between intestinal inflammatory markers and parasitic infections are scant [21–24]*.* However, a positive correlation between the level of FC and severe infections due to intestinal parasites such as *Giardia intestinalis* and *Schistosoma mansoni* has been reported [21, 22]. Conversely, lower concentrations of FC have been reported in *Blastocystis* colonized individuals compared to non-colonized subjects [25].

This study aimed to evaluate the prevalence of *Blastocystis* subtypes in HIV-positive patients, to evaluate the potential the risk factors for its transmission and the relationship between the presence of the protozoon and gastrointestinal symptoms and, finally, to assess the level of FC in *Blastocystis-*colonized subjects.

## Methods

### Study population

A cross-sectional study was carried out from January 2016 to September 2017 involving HIV-positive patients followed at the Department of Translation and Precision Medicine, Umberto I Academic Hospital, Rome. A standardized questionnaire face-to-face interview was designed including demographic data (gender, age, profession, country of origin, recent travels) and information on clinical characteristics from each participating subject (CD4+ count and HIV-RNA load, therapy, laboratory results, comorbidity), potential risk factors for intestinal pathogens (exposure to domestic animals, diet, sexual behaviour) as well as detailed gastrointestinal symptoms (diarrhoea, abdominal pain, nausea, anorexia, weight loss, weakness, flatulence). According to World Health Organization definitions, diarrhoea was defined as the passage of three or more loose or liquid stools per day*.*

Written informed consent was obtained from every participant. The study was approved with respect to the Helsinki Declaration by the Ethical Committee of the Umberto I Academic Hospital (licence n. 4836).

### Laboratory analyses

Blood and faecal samples were collected from each subject included in the study and submitted to the quantitative measurements of CD4+ T-lymphocytes and viral load [26], and to the microscopic observation of ﻿the wet smears stained with Lugol, directly or after Ridley concentration [27], respectively. Genomic DNA was then extracted from stool samples and submitted to PCR amplification using primers previously described [28], which target a fragment of about 600 bp from the *Blastocystis*-SSU rRNA gene, following PCR protocol and conditions described in Mattiucci et al., 2016 [13]. The sequences obtained were compared to those of *Blastocystis* spp. deposited in GenBank using the BLAST application (www.ncbi.nlm.nih.gov/BLAST). The STs were identified by determining the exact match or closest identity (99%), according to the classification given by Stensvold et al., 2007 [29].

Furthermore, the evaluation of the FC from a sample of subjects was performed using a commercial immunoassay (Calprest, EUROSPITAL, Italy), following the manufacturer’s instructions. Samples giving values above 50 mg/kg were regarded as having a positive Calprest test, as reported by the manufacturer and by previous published studies [30].

### Phylogenetic analyses

Bayesian inference (BI) and Neighbour-Joining (NJ) phylogenetic combined tree, based on the amplified sequences of the *Blastocystis*-SSU rDNA gene, was carried out by using MrBayes3.1 [31], software and PAUP4* [32] respectively; the analysis were performed using the HKY + G (G = 0.134) as the best model selection for the data set, as implemented in jModeltest, with Akaike Information Criterion (AIC) [33]. For Bayesian analysis, four incrementally heated Markov Chains (using default heating values), were run for 1,000,000 generations, sampling the Markov Chains at intervals of 100 generations. For each corresponding subtype, a referring sequence by GenBank database was included in the tree. *B. lapemi* and *B. pythoni* (GenBank accession no. AY590115 and KU146575, respectively*)* were used as outgroups.

### Statistical analyses

Continuous variables were summarized as mean ± standard deviation or median ± interquartile range (IQR) and categorical data as counts and percentages. Comparisons between groups were performed using χ^2^ test or Fisher’s exact test for categorical variables, and t-test or Mann-Whitney test for continuous variables. Multiple logistic regression was used to identify the predictors of *Blastocystis* colonization. The significance level for all analyses was set at *p* &lt; 0.05. Data were analysed using IBM SPSS, version 21.0 (SPSS Inc., Chicago, USA).

## Results

A total of 156 consecutive HIV-positive patients were enrolled in the study between January 2016 and September 2017. Demographic and clinical data of the study population are described in Table 1. The majority of subjects were males (75%), mean age was 47.05 ± 12.38 (range 22 to 71 years), 39 participants (25%) were coming from non-EU countries and 50 ones (32%) were MSM (*men* who have *sex* with *men*). Moreover 47.4% were owner of pets and 42.9% travelled outside Europe in the last 6 months.

**Table 1:** Epidemiological, demographic, immunologic and virological characteristics of the enrolled patients (*N* = 156). A total of *N* = 23 were naïve and *N* = 133 on ART

Study population	N (%)
Gender, male	117 (75%)
Age, years (mean ± SD)	47.05 ± 12.38
Foreign origin	39 (25%)
MSM	50 (32.0%)
Domestic animals	74 (47.4%)
Travels	67 (42.9%)
CD4 count, cells/ml, mean ± SD	655.04 ± 381.56
HIV-RNA (&lt; 37 copies/ml)	112 (71.8%)
Naïve subjects	23 (14.74%)
2NRTI + NNRTI	35 (26.3%)
2NRTI + IP/r	34 (25.6%)
2NRTI + INI	36 (27.1%)
Dual therapy	28 (21%)

Twenty-three patients (14.7%) were naïve and 133 (85.3%) were on ART treatment. Thirty-five subjects (26.3%) were on ART based on non-nucleoside reverse-transcriptase inhibitors (NNRTI), 34 (25.6%) on protease inhibitors (PI/r), 36 (27.1%) on integrase inhibitors (INI) and 28 (21%) on dual therapy. Three of the enrolled patients were on prophylaxis treatment with sulfamethoxazol-trimethoprim. The mean CD4 cell count was 655.04 ± 381.56 cells/mm^3^. Most of patients (62.2%) showed a CD4 count higher than 500 cells/ml, 26.3% patients had a CD4 count between 200 and 500 cells/ml and only 11.5% patients showed a CD4 count lower than 200 cells/ml. The median HIV-RNA level of naive patients was 40,010 (7093–824,500) -copies/mL. Among patients on ART treatment, HIV-RNA was detectable (&gt; 37 cp/mL) in 15.8% of subjects (21/133) with a median HIV-RNA level of 116 (67–991) cp/mL.

**Fig. 1:**
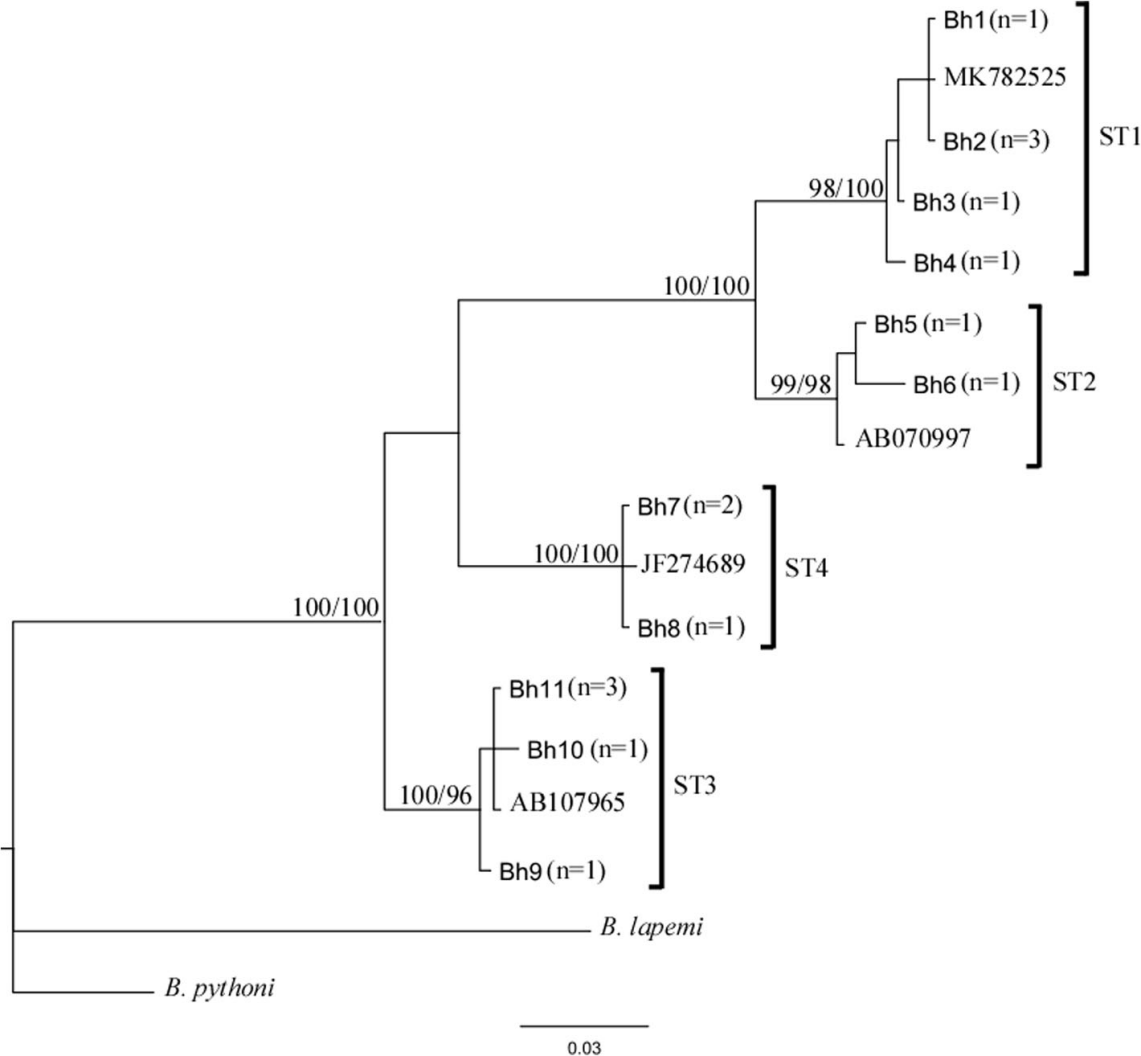
Bayesian inference (BI) and Neighbour-Joining (NJ) phylogenetic combined tree showing the relationships among *Blastocystis*-SSU rRNA gene sequences from this study (Bh) and published *Blastocystis*-subtypes ST1, ST2, ST3, ST4 (GenBank accession numbers indicated in the tree), carried out by using MrBayes3.1 and PAUP4 respectively, as above described. The number of sequences from each isolate (Bh) was reported in the brackets. Bootstrap values and posterior probabilities, respectively, are reported at the nodes; *B. lapemi* and *B. pythoni* (accession no. AY590115 and KU146575, respectively*)* were used as outgroups

Microscopic examination revealed the presence of *Blastocystis* spp. in 34 patients (21.8%) while PCR amplification detected the protist in 39 individuals (25%) ﻿and the expected 600 bp fragments of the SSU rRNA gene amplified were successfully sequenced. Sequences obtained showed a high identity (98–100%) to homologous sequences of *Blastocystis* isolates previously reported in GenBank and, according to the consensus terminology [29] and based on the phylogenetic analyses carried out, enabled the clustering of our isolates into 4 distinct *Blastocystis*-subtypes (Fig. 1).

In detail, ST3 was the most common subtype found in 51.3% of the subjects, followed by ST1 (30.8%), ST4 (10.2%) and ST2 (7.7%). As expected, ST4 was identified only in European participants. In 30.7% of the subjects, *Blastocystis* was found in coinfection with other enteric protozoa as *Entamoeba coli* (15%)*, Endolimax nana* (8%)*, Giardia intestinalis* (6.1%) and *Iodamoeba butschlii* (1.6%). The detection of such intestinal parasites was more frequent in *Blastocystis*-carriers compared to *Blastocystis*-free ones (*p* &lt; 0.0001) and in MSM compared to heterosexual subjects (*p* &lt; 0.0001).

Neither demographic characteristics such as gender, age and nationality nor other epidemiological risk factors such as travel history or, presence of domestic animals differed significantly between the *Blastocystis* positive and *Blastocystis* negative patients (Table 2). In addition, no significant differences were found between the two groups regarding CD4+ T-cell counts, HIV-RNA undetectability and type of ART regiment. *Blastocystis* positive subjects reported more frequently homosexual behavior practices compared to negative ones (48.71% vs 26.49%, *p* = 0.037). After adjusting for age, the relationship between homosexual behavior practices and the presence of *Blastocystis* remained significant (*p* = 0.01).

**Table 2:** Comparison of epidemiological, demographic, immunologic and virological characteristics between *Blastocystis*- carrier (*N* = 39) and *Blastocystis*-free (*N* = 117) patients. Significant results were marked with *

	*Blastocystis*-carriers N (%)	*Blastocystis-*free N (%)	*p*-value
Gender, male	33 (82.0%)	84 (72.6%)	0.289
Age, years (mean ± SD)	44.71 ± 11.33	47.05 ± 12.37	0.06
Foreign origin	15 (38.46%)	25 (21.36%)	0.138
MSM	19 (48.71%)	31 (26.49%)	0.037*
Domestic animals	18 (46.15%)	56 (47.86%)	0.721
Travels	20 (51.28%)	47 (40.17%)	0.296
CD4 count, cells/ml, mean ± SD	637.43 ± 263.32	660.90 ± 414.35	0.823
CD4 count &gt; 500 cells/ml	28 (71.79%)	69 (58.97%)	0.484
CD4 count 200–500 cells/ml	8 (20.51%)	33 (28.20%)	
CD4 count &lt; 200 cells/ml	3 (7.69%)	15 (12.82%)	
HIV-RNA (&lt; 37 copies/ml)	32 (82.05%)	80 (68.37%)	0.257
Naïve subjects	5 (12.82%)	18 (15.38%)	0.765
2NRTI + NNRTI	9 (23.07%)	26 (22.22%)	
2NRTI + IP/r	7 (17.94%)	27 (23.07%)	
2NRTI + INI	12 (30.76%)	24 (20.51%)	
Dual therapy	6 (15.38%)	22 (18.80%)	

Most of the *Blastocystis* positive subjects were symptomatic (53.8%), while 33.6% *Blastocystis*-free ones referred gastrointestinal disorders (*p* = 0.029). Among the gastrointestinal symptoms analyzed, flatulence was more frequently observed in *Blastocystis* carriers (30.7%) compared to *Blastocystis* negative ones (14.5%) (*p* = 0.024) (Table 3).

**Table 3:** Gastrointestinal symptoms observed in *Blastocystis*- carrier (*N* = 39) and *Blastocystis*-free (*N* = 117) patients. Significant results were marked with *

Symptoms	*Blastocystis-*carriers N (%)	*Blastocystis*-free N (%)	*p*-value
Abdominal pain	7 (17.94%)	26 (22.22%)	0.836
Diarrhoea	10 (25.64%)	20 (17.09%)	0.283
Flatulence	12 (30.76%)	17 (14.52%)	0.024*
Nausea	3 (7.69%)	6 (5.12%)	0.887
Poor appetite	5 (12.82%)	10 (8.54%)	0.180
Weight loss	3 (7.69%)	10 (8.54%)	0.658

To determine whether *Blastocystis* colonization was associated with intestinal inflammation, we measured FC in a subset of samples divided in *Blastocystis*-carrier (mono-infected) (*N* = 23) and *Blastocystis-*free (*N* = 36) subjects with comparable demographic, immunological and virological characteristics. From the all 59 fecal samples (*Blastocystis*-carrier and *Blastocystis-*free), 83% had a value in the standard range (lower than 50 mg/kg) and 11 (18.6%) had an increased value (median 115.7; 95% CI = 71.82–303.78). However, no significant differences in calprotectin values was found between *Blastocystis-*carriers (median 15, 95% CI = 18.93–148.53) and non-carriers (median 13.35, 13.93–31.74) (*p* = 0.31).

## Discussion

Studies on the prevalence of *Blastocystis* spp. in HIV-positive subjects are relatively scant. In Italy the protist has so far attracted little attention, where few epidemiological surveys have been published, demonstrating a prevalence rate of about 7% and the occurrence of seven STs (ST1, ST2, ST3, ST4, ST6, ST7, ST8) in HIV-negative people [13, 34].

The only study conducted on Italian HIV-positive patients date back to 1999, through the microscopic method, showed a *Blastocystis* prevalence of 10.3% [35]. The current study is, to our knowledge, the first to assess the prevalence of *Blastocystis* in HIV-positive patients through molecular methods in Europe and reported a prevalence of about 25%, in line with that reported in previous studies conducted in Mexico (30%) [36], Malaysia (19.8%) [15] and Iran (19%) [37]. The molecular characterization of *Blastocystis* positive isolates evidenced the circulation of 4 subtypes (ST1, ST2, ST3, ST4) already described in Italy, as previously mentioned [13, 38], being ST3 the most common one with a prevalence of 51%. In our cohort of HIV-positive patients, the presence of *Blastocystis* resulted no correlated to viro-immunological factors (*p* &gt; 0.05) confirming therefore the non-opportunistic behavior of the protist. No demographic characteristic or epidemiological drivers (presence of domestic animals, dietary habits, travels abroad) were found to be associated with *Blastocystis* colonization. A higher prevalence of *Blastocystis* was reported in MSM compared to heterosexuals as well as of the other intestinal parasites, underlying the faecal-oral contact as the main route of transmission in this group of subjects and the sexual practice and lifestyle linked to the presence of the protist more than the immunological status [16].

Despite the unresolved controversy over its pathogenicity, *Blastocystis* was also been frequently found in immunocompromised individuals presenting diarrhea [15]. In our study, among gastrointestinal symptoms, a positive association was found only for the flatulence. This socially disabling symptom is determined by two main factors: the diet, particularly the quantity of fermentable residues, and the composition and metabolic activity of colonic microbiota [39]. Since we evidenced any differences in the diet between *Blastocystis*-carriers and *Blastocystis*-free subjects, we hypothesized that this symptom was linked to the presence of the protist and the gut microbiota composition. Several Authors evaluated the intestinal microbiome in HIV-positive subjects, with somewhat inconsistent or controversial results [40]. This may be because of small sample sizes, lack of appropriate controls or regional differences in dietary and environmental factors. Regardless, ﻿many authors evidenced a change in the Bacteroides:Prevotella ratio, which they suggested to be linked to the ﻿antiretroviral therapy [41, 42] or to the ﻿sexual practice and lifestyle [43]. Similarly, several studies reported *Blastocystis* colonization associated with higher bacterial richness and Prevotella enterotype [44]. Therefore, investigations on the gut microbiota from HIV-positive subjects *Blastocystis*-carriers are needed to complete and to better understand the preliminary results obtained in this study.

Concerning FC, we found the median of both *Blastocystis*-carrier and free subjects within the normal range, supporting the non-pathogenic role of *Blastocystis* in inducing intestinal inflammation [25].

## Conclusions

Despite some critical points of this study concerning the lack of data about the faecal microbiota from enrolled subject*,* to our knowledge this is the first large survey to molecular characterize *Blastocystis* subtypes in HIV-positive patients in Europe. Our results suggest that *Blastocystis* is quite common in such patients (25%) and homosexual behavior resulted a risk factor for its transmission and confirm the presence of the protist not associated with pathological gut inflammation. The role of *Blastocystis* as intestinal pathogen remains unclear as the only symptom associated with its presence was flatulence. This result is intriguing since flatulence could be linked to the gut microbial communities, therefore the impact of the protist on the faecal microbiota and its possible role to maintain the to gut homeostasis could be interesting prospects for further studies.

## Data Availability

The dataset of this article will not be available publicly, to ensure the patient’s privacy, but are available from the corresponding author on reasonable request.

## Abbreviations

ART: Antiretroviral therapy
CI: Confidence interval
FC: Fecal calprotectin
HIV: Human Immunodeficiency Virus
INI: Integrase inhibitors
MSM: *men* who have *sex* with *men*
NNRTI: Non-nucleoside reverse-transcriptase inhibitors
NRTI: Nucleoside reverse transcriptase inhibitors
PI/r: Boosted protease inhibitor
